# *hTERT* Gene Modification Using CRISPR-dCas9-dnmt3a System as a Therapeutic Approach Against Glioma

**DOI:** 10.5812/ijpr-137226

**Published:** 2023-08-23

**Authors:** Farbod Taghavi Rad, Saied Ghorbian, Bahar Naghavi Gargari, Zeinab Shirvani Farsani, Rasoul Sharifi

**Affiliations:** 1 Department of Molecular Genetics, Ahar Branch, Islamic Azad University, Ahar, Iran; 2 Department of Genetics, School of Medicine, Shahid Beheshti University of Medical Sciences, Tehran, Iran; 3 Department of Cell and Molecular Biology, Faculty of Life Science and Technology, Shahid Beheshti University of Medical Sciences, Tehran, Iran; 4 Department of Biology, Faculty of Basic Sciences, Ahar Branch, Islamic Azad University, Ahar, Iran

**Keywords:** Glioma, CRISPR/Cas9, Telomerase, *hTERT* Gene

## Abstract

**Background:**

Abnormal DNA methylation patterns have been reported in various diseases, including different cancers. CRISPR/Cas9 is a low-cost and highly effective gene editing tool that has lately revolutionized biotechnology. Studies have shown that the CRISPR/Cas9 system can effectively target and correct methylation.

**Objectives:**

Telomerase plays a survival role for cancer cells. It is encoded by the *hTERT* gene. The effectiveness of CRISPR/Cas9 in targeting hTERT to treat glioma cancer cells was assessed in this study.

**Methods:**

EF1a-hsaCas9-U6-gRNA vector carrying sgRNA and Cas9 hybrids were used to transfect U87 glioma cells. Four and eight μg/mL polybrene concentrations were investigated to improve transfection efficiency. The expression level of *hTERT* that has undergone metabisulfite modification was assessed using real-time PCR. Flow cytometry and Western blotting were also used to determine whether telomerase was present in the cells. High-resolution melting analysis (HRM) was used to examine the *hTERT* promoter's methylation. Finally, flow cytometry was used to measure the apoptotic rate of transfected U87 cells.

**Results:**

The findings demonstrated that gRNA significantly boosted transfection effectiveness. Significant variations were seen in the expression of *hTERT* in U87 cells at 4 μg/mL polybrene and 80 μg/mL transfection compared to transfection without gRNA and basal cells. Flow cytometry showed a decrease in *hTERT* levels in transfected cells. Furthermore, transfection with gRNA increased U87 cell apoptosis compared to transfection without gRNA.

**Conclusions:**

It appears that the designed CRISPR/Cas9 system can reduce *hTERT* expression and telomerase activity and thus inhibit glioma cell growth.

## 1. Background

The most prevalent and severe brain cancer in adults is glioma (1). The kind, location, and severity of the tumor and the patient's general health determine the treatment options for acute glioma (2). Poor treatment and frequent recurrence of glioma result from the blood-brain barrier, which limits the entry of chemical medications into the brain, and the tumor's recurrence is due to the self-renewal of glioma stem cells. The average survival time for glioma patients has decreased due to this phenomenon (3).

Cancer develops when cells accumulate genetic mutations over time. Additionally, some genetic modifications, such as driver mutations, can create cancer cells (4). The majority of the time, treatment plans are based on histology subgroups. However, in addition to the traditional histological categories, each form of cancer can now be further broken down into many molecular subgroups that are crucial in the treatment choice (5). Medical professionals can forecast treatment outcomes and patient survival and treat each genomic subtype differently. As a result, treatment plans are changed in accordance with new molecular markers. Therefore, identifying new treatment options is critical for improving patient survival and clinical outcomes (6).

Telomerase is rapidly activated in germ cells, hematopoietic cells, stem cells, as well as highly reproducible cells. In contrast, telomerase activity in somatic cells is very low, mostly due to the strong regulation of *hTERT* (7). A deeper knowledge of the underlying mechanisms of *hTERT* regulation has recently gained momentum, mostly due to advancements in the detection of *hTERT* promoter mutations. Over the past 20 years, researchers have studied the processes that control *hTERT*. Nevertheless, additional alterations have been found and shown to increase *hTERT* expression through a number of genetic and epigenetic mechanisms, such as *hTERT* amplification, *hTERT* structural mutations, *hTERT* promoter mutations, and epithelial alterations genetically regulated by *hTERT* promoter methylation (8).

Although some researchers have demonstrated hypomethylation in the CpG islets surrounding the *hTERT* promoter, others have reported increased DNA methylation in cancer cells that express *hTERT* (9). As a matter of fact, *hTERT* was among the first genes in which promoter methylation and gene expression had a favorable association. This association between the *hTERT* promoter's methylation and telomerase activity and *hTERT* mRNA shows that the *hTERT* promoter may regulate this gene, but not in the same manner that promoter methylation regulates other genes (10).

Biotechnology and genetic engineering have undergone a significant transformation in recent years thanks to practical and affordable gene editing technologies like CRISPR/Cas9. Genomic alterations can be targeted and employed to cure hereditary illnesses using the CRISPR/Cas9 system (11). Methylation modification is one method of gene editing available with the CRISPR/Cas9 system. The DNMT enzyme family is responsible for DNA methylation (12). Human DNA methyltransferase's catalytic domain performs an enzymatic role in transfected cells, making DNMT3A the most active isoform (13).

## 2. Objectives

In this study, the main promoter of the *hTERT* gene and its expression in the U87 cell line for malignant gliomas were targeted using CRISPR/Cas9 system.

## 3. Methods

### 3.1. Plasmid Vector Preparation

**Figure 1.**
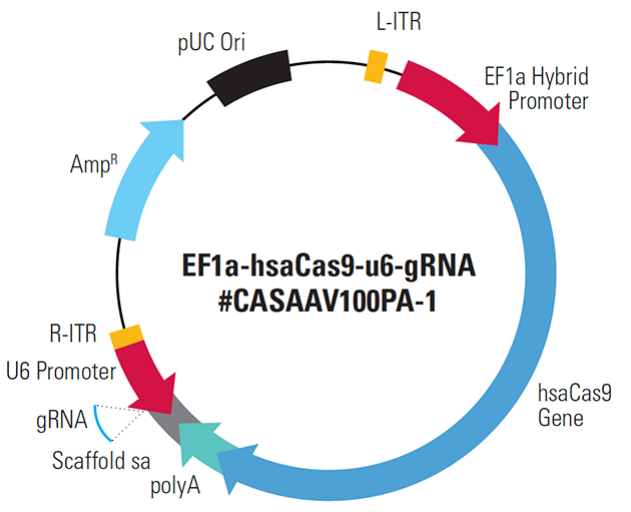
The schematic of the designed plasmid

CRISPR software was used to design the sgRNA sequence, and the result was CCAGGACCGCGCCTTCCCACG. Using a system biosciences (SBI) kit, the plasmid vector was created. EF1a-hsaCas9-U6-gRNA (#CASAAV100PA-1) vector (Figure 1), a hybrid sgRNA and Cas9 carrier, was also employed. The sgRNA fragment was found between the promoter of the U6 gene and the poly-A region of the *hsaCas9* gene.

### 3.2. Cell Preparation and Culture

The Iranian National Centre for Genetic and Biologic Resources provided the glioma cell line U87. U-87 is a cell line with epithelial morphology isolated from malignant gliomas from a male patient with a possible diagnosis of glioblastoma. These cells were cultured in the AmnioMAX culture medium (Gibco-America), which was incubated at 37.4°C and 5% CO_2_.

### 3.3. Transfection Process

The Lipofectamine CRISPRMAX kit was used to transfect U87 cells. The cells were cultured for this purpose in an RPMI culture medium with 15% FBS. The transfection effectiveness was increased by adding two concentrations of 4 and 8 µg/mL hexadimethrine bromide (polybrene) to each well after the cells had reached a confluency and density of 30 - 70%. After 48 hours, the wells' media were removed, and RPMI culture medium with 15% FBS and concentrations of 4 and 8 µg/mL polybrene was added. This mixture was then incubated for 30 minutes. The following step involved combining 10^3^ cells, 50 ng of gRNA (80 and 160 µg/mL), 250 ng of Cas9 nuclease, 0.5 μL of Cas9 Plus solution, and 5 μL of Opti-MEM I medium. After that, 0.3 μL of CRISPRMAX was dissolved in 5 μL of Opti-MEM I and immediately added to the earlier formulations. A volume of 10 μL of this mixture was applied to each well containing U87 cells after they had been incubated for five to ten minutes. To prepare cell lysates, the cells were cultured for two to three days at 37°C before the culture medium was removed. The cells were then washed with 50 to 500 μL of PBS and lysed with 50 to 250 μL of lysis buffer.

### 3.4. Detection of Cas9 in Transfected Cells

In the transfected cells, Cas9 was found using flow cytometry. After 24-hour transfection, on average, 10^3^ cells were put into each well for this purpose. After 72 hours of incubation, the cells were removed from the plate and centrifuged after being fixed with PBS and one unit of the trypsin enzyme. The cell pellet was washed in a 10% binding buffer solution, and centrifugation was then carried out for 15 minutes at 15000 rpm. In the end, a flow cytometer was used to conduct the reading.

### 3.5. Evaluation of DNA Methylation and hTERT Gene Expression

For *hTERT* gene methylation, the EZ-96 DNA Methylation-Gold™ kit (Zymo Research, Irvine, CA) was used according to the manufacturer's instructions. A concentration of 0.05 μL of metabisulphite-modified DNA was considered as a template for real-time PCR. According to the kit instructions, the amplification reaction was carried out in a volume of 20 μL and included 10 μL of the master solution (Master Mix), 0.4 μL of each of the forward (5’-CACGCGAAAACCTTCCTCAG-3’), and reverse (5’-GGCCTCGTCTTCTACAGGGA-3’) primers, 5 μL of metabisulphite-modified DNA, and 4.2 μL of distilled water. The eva-green real-time RT-PCR kit, Biotium (South Korea), was used for this reaction. 10 μL of SYBR Green, 2 μL of cDNA sample, 0.5 μL of forward and reverse primers (10 pmol) were mixed with 7 μL of nuclease-free water (Qiagen, Hilden, Germany). The *GAPDH* housekeeping gene was used as a reference, and *hTERT* gene expression was normalized using it. Evaluation of *hTERT* methylation and expression was carried out in cells transfected without gRNA, transfected with gRNA, as well as basal cells as a positive control.

### 3.6. Investigation of Telomerase Activity in Cells by Western Blot Technique

To perform western blot, the microtube containing cells was centrifuged at 1300 rpm for 10 minutes, and after discarding the supernatant, 100 µL of RIPA buffer (10 mM Tris-Cl pH 8.0, 1 mM EDTA, 0.5 mM EGTA, 1% Triton X-100, 0.1% sodium deoxycholate, 0.1% SDS, and 140 mM NaCl) was added to the cell pellet. Following storing for 1 hour at -20°C, the falcon was placed at room temperature for 5 minutes and then centrifuged at 1300 rpm for 10 minutes at 4°C. Then the protein-containing supernatant was transferred to a new microtube. The SDS-PAGE gel in a single electrophoresis run was divided into stacking gel and separating gel. The stacking gel (acrylamide 5%) was poured on top of the separating gel, and a gel comb was inserted into the stacking gel. The protein isolated by SDS-PAGE was transferred to the PVDF membrane, which was blocked with a blocking solution (5% skim milk in 0.05% PBS-Tween 20) at -4°C for 12 hours. Then washing with PBS-Tween 20 was performed three times for 5 minutes, and it was incubated for 90 minutes at 25°C with 10 µg/mL monoclonal anti-*hTERT* antibody (Padza Co, Iran). The membrane was washed three times with PBS-Tween 20 for 5 minutes and exposed to 0.4 µg/mL secondary HRP antibody (Padza Co, Iran) for one hour at 25°C. After washing the membrane, the bands were detected using a gel documentation system.

### 3.7. Evaluation of Telomerase Activity in Cells Through Flow Cytometry

Telomerase protein levels were assessed using flow cytometry. The telomerase levels were assessed in cells transfected with gRNA, cells transfected without gRNA, and basal cells acting as a positive control using the same technique. For this purpose, an average of 10^3^ cells were placed in each well in a 24-well plate. After incubating for 24 hours, the cell sediment was obtained using the cell passage method. A volume of 250 µL of a solution containing propidium iodide, according to the instructions of the Biolegend kit and Triton X100, was added to the resulting cell sediments. Cells were incubated in darkness for about an hour. The cells were then read immediately using a flow cytometry device, and telomerase level was obtained by comparison with the control group.

### 3.8. Evaluation of hTERT Gene Promoter Methylation

Gene methylation was evaluated using an high-resolution melting (HRM) assay. A 40-cycle-replication reaction was performed. For this purpose, enzyme activation (hot start) was performed at 95°C. The cycle began with an initial denaturation and holding period at 95°C for 10 seconds, followed by 20 seconds of annealing at 57°C. It concluded with a 25-second extension at 72 degrees. The temperature was also conducive to light absorption. Following these procedures, a melting step was carried out at a temperature range of 55 to 95°C to separate the DNA bands impacted by metabisulfite using HRM. The forward and reverse primers were in the following order: 5’-GCAATGCGTCCTCGGGTTC-3’ and 5’-CGGAGAGAGGTCGAATCGG-3’, respectively. The methylation percentage was then calculated from the ratio of heights of a cytosine peak (methylated signal) and the sum of cytosine and thymine peaks (methylated and unmethylated signal) for each cytosine in a CpG dinucleotide.

### 3.9. Evaluation of Apoptosis

Flow cytometry was used to evaluate the apoptosis rate of U87 cells. In each well of a 24-well plate, 10^3^ cells were cultured for this purpose, and after 72 hours, the level of apoptosis was determined. The cells were taken off the plate and centrifuged after being cleaned with PBS and one unit of the trypsin enzyme. The cell pellet was then centrifuged at 15000 rpm for 15 minutes after being rinsed with a 10% binding buffer solution. A volume of 5 μL of the Annexin V-FITC solution was incubated for 15 minutes at room temperature. Then they were prepared using 5 μL of propidium iodide and counted by flow cytometer. The results were analyzed using FlowJo software.

### 3.10. Statistical Analysis of Data

All experiments were carried out three times, and the results were computed as the mean ± standard deviation. The SPSS V.22 software was used for data analysis. The one-way ANOVA approach was used to compare the treatment and control groups, taking into account the normal distribution of the data, and the P-value was obtained. The threshold for significance was set at P < 0.05. A post hoc test was regarded as Tukey's test.

## 4. Results

### 4.1. Confirmation of the Presence of Cas9 Using Flow Cytometry

**Figure 2.**
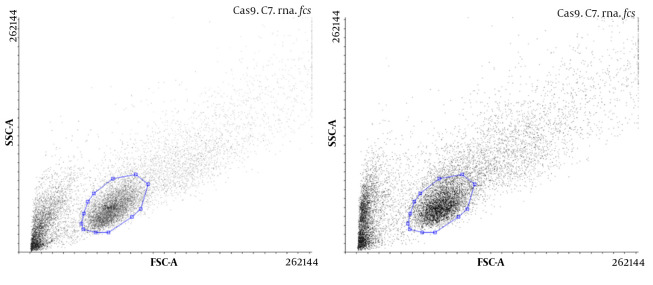
The presence of Cas9 in U87 cells transfected without gRNA (A); and with gRNA (B) using flow cytometry, which shows an increase in transfection efficiency in the presence of gRNA.

The presence of Cas9 was evaluated, and the transfection efficiency was compared with and without gRNA in U87 cells using flow cytometry. The results showed that Cas9 was present in both types of transfected cells, but in the presence of Cas9, gRNA transfection was more efficient (Figure 2). 

### 4.2. Evaluation of hTERT Gene Expression in U87 Cells

**Figure 3.**
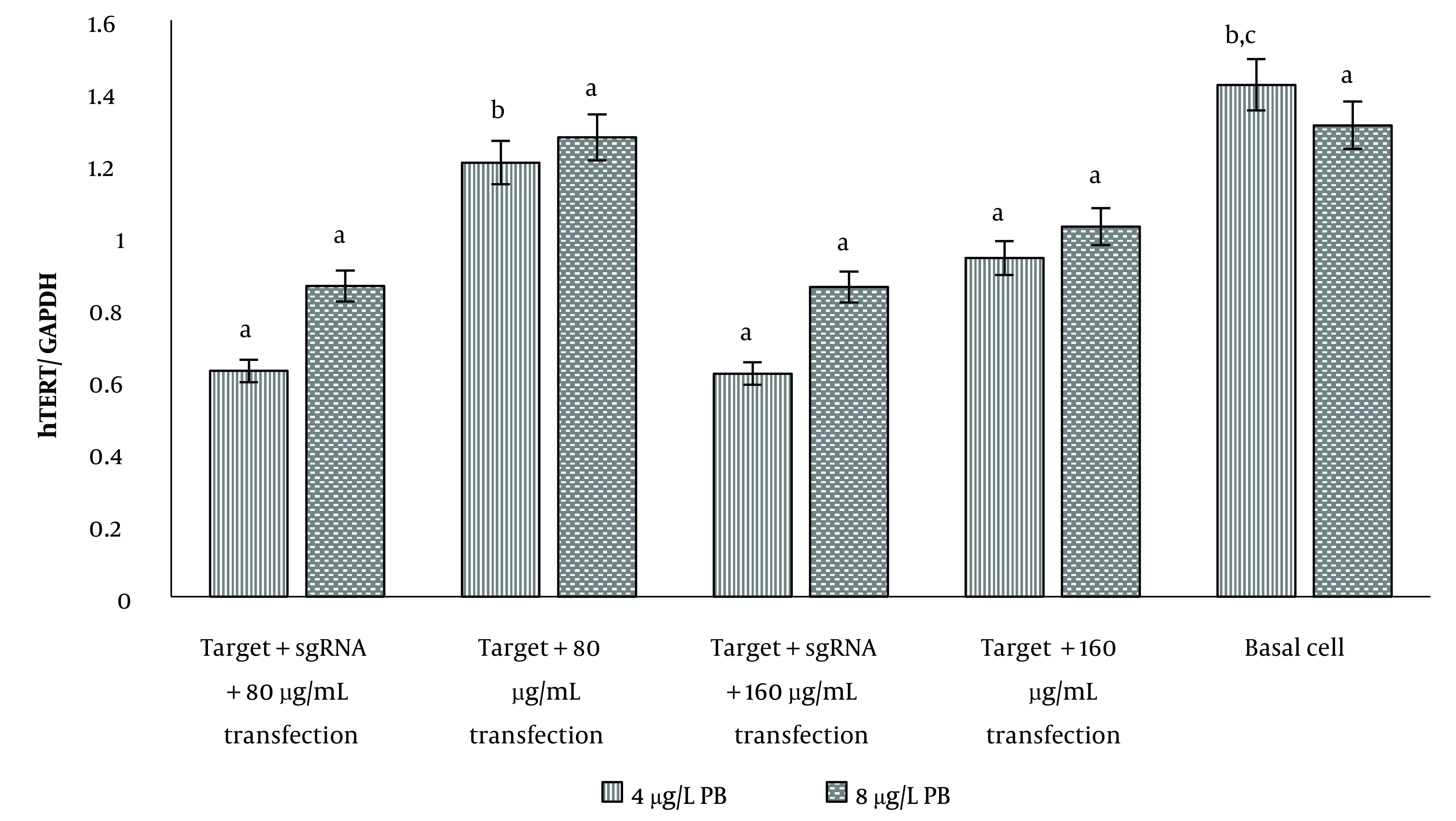
Comparison of *hTERT* gene expression in transfected U87 cells in the presence of 4 and 8 μg/mL of polybrene. a, no significant difference with controls; b, significant difference with 80 μg/mL transfection control; c, significant difference with 160 μg/mL transfection control

The *hTERT* gene expression in U87 cells showed that only at a transfection concentration of 80 μg/mL of gRNA, expression of this gene was significantly reduced compared to the case without gRNA. At the transfection concentration of 160 μg/mL of gRNA, no difference was observed between gene expression in the presence and absence of gRNA (Figure 3). In both transfection concentrations, *hTERT* expression showed a significant decrease compared to basal cells (P < 0.05). In transfected cells with 8 μg/mL polybrene, there was no significant difference in *hTERT* gene expression in the studied groups, including gRNA-transfected cells, non-transfected cells, and basal cells (Figure 3). 

### 4.3. Evaluation of hTERT Protein Level

**Figure 4.**
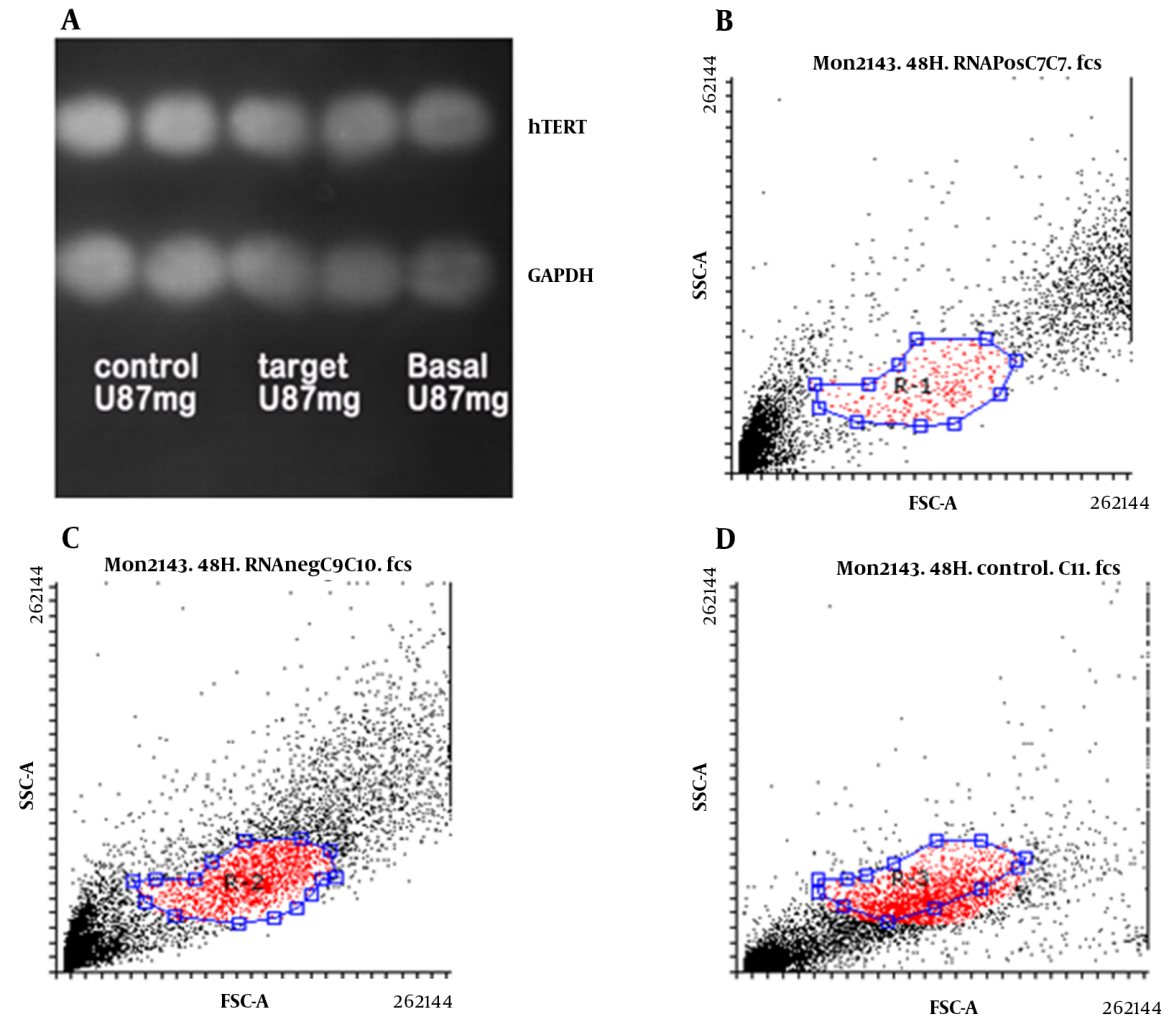
*hTERT* protein level in U87 cells transfected with gRNA (target) and without gRNA (control) as well as basal cells compared to GAPDH protein level by Western blotting (A); and flow cytometry in basal cells (B); transfected U87 cells without gRNA (C); and transfected U87 cells with gRNA (D)

Using the Western blotting method, *hTERT* protein level was measured in U87 cells transfected with and without gRNA as well as basal cells relative to GAPDH protein. As shown in Figure 4A, no significant difference was observed in the amount of *hTERT* protein in U87 cells in the studied cells. However, examination of *hTERT* protein levels in U87 cells using flow cytometry revealed that this protein was higher in the basal cells (Figure 4B) and transfected cells without gRNA (Figure 4C) than in the transfected cells with gRNA (Figure 4D). 

### 4.4. Evaluation of hTERT Gene Methylation

**Figure 5.**
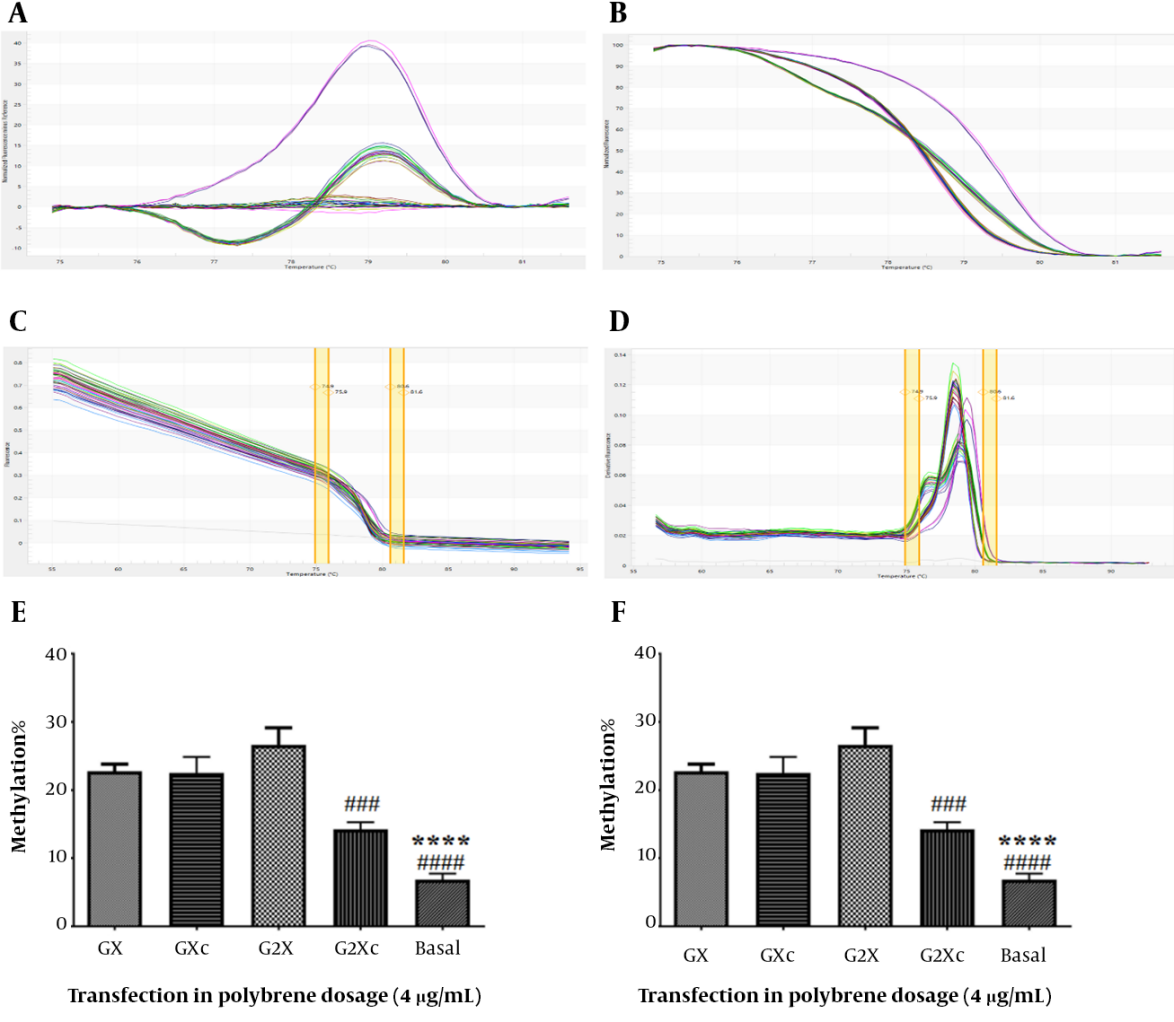
Evaluation of *hTERT* gene methylation. A, difference melting curve; B, normalized melting curve; C, component melting curve; D, derivative melting curve; E, *hTERT* gene promoter methylation in transfected U87 cells in the presence of 4 μg/mL polyburn; F, methylation of the *hTERT* promoter in transfected U87 cells in the presence of 8 μg/mL polybrene. GX: Target + sgRNA + 80 μg/mL transfection, GXc: Control + 80 μg/mL transfection, G2X: Target + sgRNA + 160 μg/mL transfection, G2Xc: Control + 160 μg/mL transfection. **: Significant difference with GX group with P < 0. 01, ****: Significant difference with GX group with P < 0.0001, #: Significant difference with G2X with P < 0.05, ###: Significant difference with G2X with P <0.001, ####: Significant difference with G2X with P < 0.0001

*hTERT* gene methylation was assessed using the HRM method (Figure 5A-D). In U87 cells at a concentration of 4 μg/mL polybrene, it was shown that only at the transfection concentration of 160 μg/mL methylation of the *hTERT* promoter in the presence of gRNA was significantly increased compared to its absence (Figure 5E). Compared with basal cells, both 80 and 160 μg/mL transfection concentrations significantly increased *hTERT* promoter methylation (P < 0.05). At a concentration of 8 μg/mL polybrene, both 80 and 160 transfection concentrations significantly increased the methylation of the *hTERT* promoter in the presence of gRNA relative to its absence in U87 cells (Figure 5F). Also, a significant increase in *hTERT* promoter methylation was observed in both 80 and 160 μg/mL transfection concentrations compared to basal cells (P < 0.05).

### 4.5. Evaluation of Apoptosis Rate in U87 Cells

**Figure 6.**
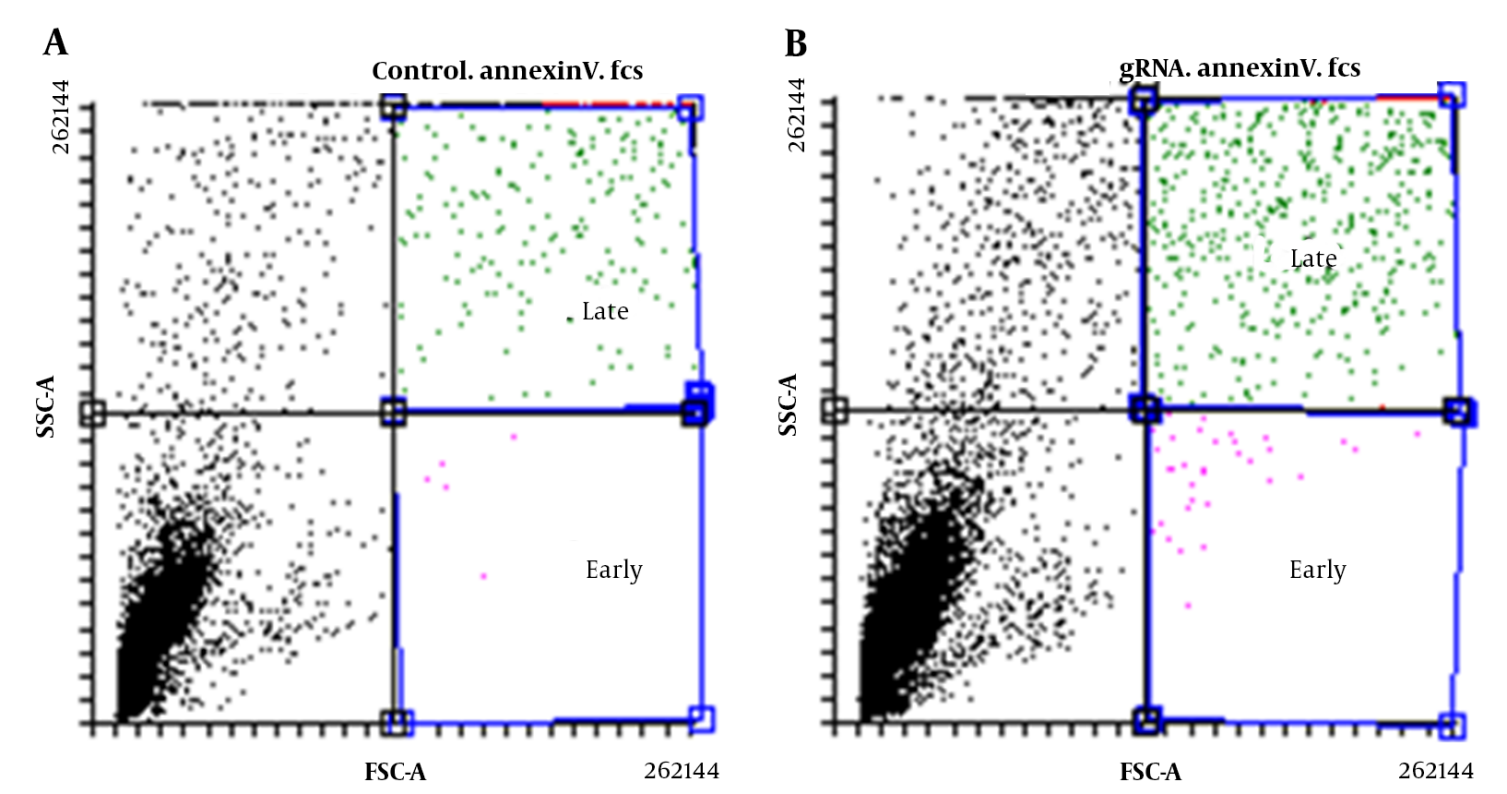
Flow cytometry to investigate cell death in transfected U87 cells without gRNA (A); and with gRNA (B)

Flow cytometry was used to assess the apoptosis rate in transfected U87 cells, and the results showed both early and late apoptosis rates were higher in transfected cells with gRNA compared to transfected cells without gRNA (Figure 6). In fact, in the absence of gRNA, the amount of early and late apoptotic cells was estimated to be 0.06% and 7.01%, respectively, while in the presence of gRNA, these values reached 0.27 and 11.37%, respectively (P < 0.05).

## 5. Discussion

The results of the present study indicated that in U87 cells, only at a concentration of 4 μg/mL polybrene and 80 μg/mL transfection, there was a significant difference in the expression of *hTERT* compared to transfection without gRNA and basal cells. Evaluation of *hTERT* promoter methylation also showed that in U87 cells at concentrations of 4 and 8 μg/mL polybrene and 80 and 160 μg/mL transfection, there was a significant increase in *hTERT* promoter methylation compared to basal cells. The level of *hTERT* protein in U87 cells showed an increase only in flow cytometry results. Nevertheless, Western blotting did not reveal any appreciable variations in the levels of *hTERT* protein in the cells. Finally, it was shown that transfection with gRNA in U87 cells increases the induction of apoptosis compared to transfection without gRNA. The present study's findings generally demonstrated that the designed CRISPR/Cas9 system could kill U87 glioma cancer cells by decreasing the expression of *hTERT* at the RNA and protein levels and by increasing the methylation of its promoter.

Cancer occurs when genomic instability accumulates in normal cells, and the cells reach limitless proliferation capacity. Cancer cells can delay the effects of aging by activating telomerase or using other mechanisms to prolong the length of their telomeres. In 1994, it was reported that telomerase controls 90% of malignant tumors, which is essential for the development of cancer and other diseases (14, 15). Although repetitive mutations in the *hTERT* promoter play a significant role in activating *hTERT* in malignancies, there are other tumor types (such as prostate and breast cancer) where these mutations are less common (16). Because of this, the importance of epigenetic mechanisms in regulating *hTERT* differs depending on the kind of cancer, and many studies have shown different results in terms of the impact of *hTERT* promoter methylation on *hTERT* regulation. Promoter methylation and gene silencing usually occur together. However, many investigations have shown that methylation of certain regions within the *hTERT* promoter, particularly upstream of the main promoter, is associated with gene activation (14). The specific mechanisms by which the pattern of *hTERT* promoter methylation results in *hTERT* activation are still being investigated. Recently, it has been shown that *hTERT* promoter methylation may play a role in activating the expression of *hTERT* (17).

There are various possibilities as to how *hTERT* activation can result from *hTERT* promoter methylation. Based on the prevention of suppressor elements binding in the repressor region, the first hypothesis is a DNA methylation-related mechanism. If the *hTERT* promoter is hypomethylated (or non-methylated), transcription inhibitors bind to the promoter and block transcription machines. If *hTERT* is methylated, though, it prevents this binding and makes it possible for the promoter to be turned on by proper transcription factors. Interesting observations have revealed that the upstream region of the primary promoter of *hTERT* is frequently hypermethylated, thus preventing the key stimulus of gene expression from accessing the promoter. Another explanation is more complicated and involves chromosome structural alterations and DNA methylation. By altering the structure of chromatin and the impact of DNA exposure on transcription factor binding, DNA methylation can influence gene expression (18). Histone modifications and DNA methylation are frequently linked, and the latter may regulate how transcription factors can access the promoter. Specific conformational changes caused by *hTERT* promoter methylation may result in different levels of uptake and binding of factors that may cause *hTERT* overexpression in cancer (19). Histone acetylation and methylation are two post-translational histone changes that can influence chromatin density and, consequently, gene expression (20).

Malignant blood, prostate, urothelium, brain, and colon tumors, among other types of cancer, have all shown a high frequency of hypermethylation in the region upstream of the major *hTERT* promoter. More surprisingly, *hTERT* promoter methylation is linked to *hTERT* overexpression even in melanoma, where *hTERT* promoter alterations were originally discovered, and a mechanism for *hTERT* activation was considered (21). Although this tumor-specific signature is found in a wide variety of tumor types, more work needs to be done to translate these findings to clinical settings. Pediatric glioma possesses a methylated area in the *hTERT* promoter that may serve as a potential biomarker for tumor survival and progression (14). Malignant tumors have hypermethylation in this region, known as THOR, while healthy tissues and stem cells have hypomethylation. Prostate cancer research has focused on THOR and has demonstrated its potential as a marker with diagnostic and prognostic properties (17).

Gigek et al. (22) examined the methylation and expression of *hTERT* in gastric cancer and demonstrated that *hTERT* was not expressed in healthy individuals, while in 80% of tumor tissues, this gene was expressed. In contrast, the hypermethylation of the *hTERT* promoter in tumor samples was significantly higher than in healthy samples. However, they reported that there was no significant relationship between promoter hypermethylation and *hTERT* expression (22). Bougel et al. (23) reported that in the cerebrospinal fluid of people with metastatic leptomeningeal cancer, approximately 1% of *hTERT* was methylated, and the rest was non-methylated and that the methylation of the *hTERT* promoter can be used as a diagnostic marker. Modification of methylation and expression of *hTERT* has been reported in different types of cancers (24-26), and most stated that with increasing methylation, the expression of *hTERT* increases. However, in the present study, it was shown that increased methylation was associated with the downregulation of *hTERT*. In this respect, one can say that the type of cancer studied can affect the observed mechanisms.

This work exploited the activation of *hTERT* promoter methylation as an epigenetic alteration to develop a treatment for glioma malignancy. Most epigenetic modifications, in contrast to gene mutations, may be reversed or prevented. In the treatment or prevention of cancer, it has become increasingly popular to restore abnormal epigenetic events in neoplastic cells (27). Before the development of CRISPR technology, scientists used a variety of instruments that could separate DNA double strands to modify the genome in various ways. As an example, four different subcategories of DNA-binding nucleases, such as Cas9 nuclease, the most modern meganucleases, zinc finger nucleases (ZFNs), and transcription activator-like effector nucleases (TALEN), were considered. Every known nuclease has its restrictions. Zinc finger nucleases contain distinct DNA-binding domains as well as the Fok1 endonuclease, a generic cleavage domain. The main difficulty also lies in the design and building of ZNFs (28). Low binding to and separation from the target sequence is another drawback of this method, which reduces its effectiveness (29). We used the potent CRISPR technology in this study because it was a much cheaper and simpler way to make various changes to the genome.

In a prior investigation, the authors used the A-375 cell line connected to malignant melanoma of pseudoepithelial origin to analyze the methylation alterations of the major promoter of the *hTERT* gene and the amount of its expression. They used dCas9, a modified version of Cas9, and DNMT3A, a similar protein. The outcomes demonstrated that the developed CRISPR/Cas9 system decreases *hTERT* expression and telomerase, and thus it can prevent melanoma cells’ growth (30).

The PTEN gene was chosen by Moses et al. (31) as the target of the CRISPR/Cas9 system in the SK-MEL-28 melanoma cell line, and it was demonstrated that this repair system is capable of upregulating PTEN and reducing cancer cell migration. The main reason for their observations, as they stated, was decreased signaling of oncogenic pathways such as AKT, mTOR, and MAPK. In a related study, Moses et al. (31) reported that the CRISPR/Cas9 gene modification in the melanoma cell line resulted in an upregulation of PTEN. Their research used the PI3K/mTOR pathway's inhibition as the repair system's mechanism of action. In relation to glioma, studies have been used to use the CRISPR/Cas9 gene editing system to inhibit cancer cells, but according to the author's search in the databases, none of them was on the *hTERT* gene. Huang et al. (32) used the CRISPR/Cas9 system to target exon 17 of the *EGFR* gene in glioma cells, thereby activating the inhibitory modification of *UBXN1* and inhibiting tumor necrosis factor in these cells. Using the CRISPR/Cas9 system, Morimoto et al. (33) inhibited the *TIM3* gene in natural killer cells, thereby inhibiting the growth of glioma cells. Moure et al. (34) used the CRISPR/Cas9 editing system to knock out the *IDH1* and induce CpG demethylation in glioma models. Therefore, it seems that the CRISPR/Cas9 gene editing system can be well used to inhibit the growth of cancer cells, especially by targeting the methylation status of genes, and in various studies, as mentioned, this system has been used to modify the methylation and expression of genes involved in glioma. Target genes and methylation and demyelination status can differ depending on the type of cancer's target mechanisms. What was observed in the present study was that the CRISPR/Cas9 system in glioma can downregulate this gene by increasing the promoter methylation of the *hTERT* gene, thereby killing cancer cells.

### 5.1. Conclusions

Since the development of genomic editing tools, CRISPR/Cas9 technology has revolutionized biology by facilitating the modification of the genomes, transcriptomes, and epigenomes of different organisms. Cancer research could be revolutionized by CRISPR/Cas9, but this has not yet been completely realized. This technique was employed in this study for methylation, and the outcomes showed how well this technology led to apoptosis in U87 cells. In fact, the treatment of glioma produced positive results thanks to the employment of CRISPR/Cas9 as a therapeutic option and the *hTERT* gene as a therapy target. In the future, CRISPR technology may target various genes in many cancer cells. CRISPR can spot deadly cancer interactions and help find novel therapeutic targets by combining genomic and epigenetic data from cancer cell lines.

## Data Availability

The dataset presented in the study is available on request from the corresponding author during submission or after publication.
